# The Effect of Modified Porcine Surfactant Alone or in Combination with Polymyxin B on Lung Homeostasis in LPS-Challenged and Mechanically Ventilated Adult Rats

**DOI:** 10.3390/molecules25194356

**Published:** 2020-09-23

**Authors:** Maros Kolomaznik, Jana Kopincova, Zuzana Nova, Juliana Topercerova, Ivan Zila, Pavol Mikolka, Petra Kosutova, Katarina Matasova, Henrieta Skovierova, Marian Grendar, Daniela Mokra, Andrea Calkovska

**Affiliations:** 1 Biomedical Centre Martin, Jessenius Faculty of Medicine in Martin, Comenius University in Bratislava, 03601 Martin, Slovakia; maros.kolomaznik@uniba.sk (M.K.); pavol.mikolka@uniba.sk (P.M.); petra.kosutova@uniba.sk (P.K.); henrieta.skovierova@uniba.sk (H.S.); marian.grendar@uniba.sk (M.G.); 2 Department of Physiology, Jessenius Faculty of Medicine in Martin, Comenius University in Bratislava, 03601 Martin, Slovakia; janka.kopincova@gmail.com (J.K.); zuzanka.nova@gmail.com (Z.N.); jtopercerova@gmail.com (J.T.); Ivan.Zila@jfmed.uniba.sk (I.Z.); daniela.mokra@uniba.sk (D.M.); 3 Clinic of Neonatology, Jessenius Faculty of Medicine in Martin, Comenius University in Bratislava and Martin University Hospital, 03601 Martin, Slovakia; matasova.katka@gmail.com

**Keywords:** ARDS, bacterial lipopolysaccharide, pulmonary surfactant, surfactant proteins

## Abstract

The study aimed to prove the hypothesis that exogenous surfactant and an antibiotic polymyxin B (PxB) can more effectively reduce lipopolysaccharide (LPS)-induced acute lung injury (ALI) than surfactant treatment alone, and to evaluate the effect of this treatment on the gene expression of surfactant proteins (SPs). Anesthetized rats were intratracheally instilled with different doses of LPS to induce ALI. Animals with LPS 500 μg/kg have been treated with exogenous surfactant (poractant alfa, Curosurf^®^, 50 mg PL/kg b.w.) or surfactant with PxB 1% w.w. (PSUR + PxB) and mechanically ventilated for 5 hrs. LPS at 500 μg/kg increased lung edema, oxidative stress, and the levels of proinflammatory mediators in lung tissue and bronchoalveolar lavage fluid (BALF). PSUR reduced lung edema and oxidative stress in the lungs and IL-6 in BALF. This effect was further potentiated by PxB added to PSUR. Exogenous surfactant enhanced the gene expression of SP-A, SP-B, and SP-C, however, gene expression for all SPs was reduced after treatment with PSUR + PxB. In mechanically ventilated rats with LPS-induced ALI, the positive effect of exogenous surfactant on inflammation and oxidative stress was potentiated with PxB. Due to the tendency for reduced SPs gene expression after surfactant/PxB treatment topical use of PxB should be considered with caution.

## 1. Introduction

Bacterial endotoxin (lipopolysaccharide, LPS) is the major component of the outer membrane of Gram-negative bacteria [1]. In the respiratory system, it binds to the toll-like receptor (TLR) complex CD14/TLR4/MD-2 on cellular membranes [2]. It also interacts with the pulmonary surfactant, a lipoprotein material lining the inner surface of the lung, which is an important component of the innate host defense against invading pathogens [3]. Activation of TLRs leads to an increase in transcription factor NF-κB, activator protein 1, and interferons, and induces both pro-inflammatory and pro-oxidative pathways [4] which may ultimately result in acute respiratory distress syndrome (ARDS). Due to the high morbidity and mortality of ARDS, new possibilities in the treatment are being intensively sought [5]. While in neonatal RDS of prematurity exogenous surfactant replacement is routinely used, in ARDS due to the complex pathophysiology this treatment has failed in clinical trials [6]. Plasma proteins, inflammatory mediators, or LPS present in the alveolar space lead to surfactant inhibition which can be reversed by substances improving its biophysical and physiological characteristics.

Polymyxin B (PxB) is the antimicrobial peptide from the bacterium *Bacillus polymyxae* mainly used to treat Gram-negative infection [7]. In addition to its antibiotic effect, due to its property to bridge and stabilize phospholipid membranes, it is able to mimic the functions of the surfactant protein B [8]. PxB has no adverse effect on the biophysical activity of the surfactant and moreover, improves the resistance of surfactant to albumin [9], meconium, and lipopolysaccharide [10] in vitro.

Data on nephrotoxic and neurotoxic properties of polymyxins published several decades ago have been revisited. This group of antibiotics appears to be less toxic than previously reported [11], but there is still concern about their possible adverse effects on systemic administration. Therefore, topical administration, for example in the mixture with an exogenous surfactant as a carrier, enhances the delivery of these drugs to the deeper regions of the lung [12] and appears to be a suitable alternative. In mixture with a surfactant, it retains its bactericidal properties and reduces entry *Escherichia coli* (*E. coli*) from the alveolar space into the bloodstream in neonatal models of pneumonia [13]. Encouraging results were recently obtained in a similar pneumonia model with polymyxin E, where the mixing of polymyxin with surfactant increased its bactericidal effect. This was explained by more efficient spreading caused by interactions between polymyxin E and surfactant [14].

Even topical lung administration may not be safer as indicated by recent in vitro studies. Recently, it was demonstrated that both extrinsic death receptors and intrinsic mitochondrial pathways are involved in polymyxin-induced lung toxicity [15], and intracellular localization of polymyxins in human alveolar epithelial cells A549 was identified [16]. PxB reduced viability and stimulated exocytosis in alveolar type II (ATII) cells isolated from rat lungs. Mixing polymyxin B with pulmonary surfactant hampered the negative effect of PxB on vitality and surfactant exocytosis [17]. The reduction in cellular toxicity is explained by the binding of PxB to the surface film allowing the connection between the two phospholipid monolayers, thereby slowing down the release of PxB from the surfactant lipoprotein complex and reducing its maximum concentration in the cell.

The concept of the possible use of exogenous surfactant for the delivery of antimicrobial agents including polymyxins is widely discussed. The results so far are encouraging, however, the effect of such a mixture on lung tissue at the molecular level is almost unknown.

The aim of the study was to prove the hypothesis that therapy with surfactant/polymyxin B is more effective in reducing the LPS-induced lung injury than surfactant treatment alone in a rat model of LPS-induced lung injury. Since surfactant proteins SP-A, SP-B, SP-C, and SP-D are expressed at relatively high levels in ATII cells [18] and are crucial for alveolar homeostasis, another objective was to evaluate the impact of this treatment on the expression of genes encoding the surfactant proteins.

## 2. Results

### 2.1. First Series

#### 2.1.1. Lung Oedema Formation

**Figure 1:**
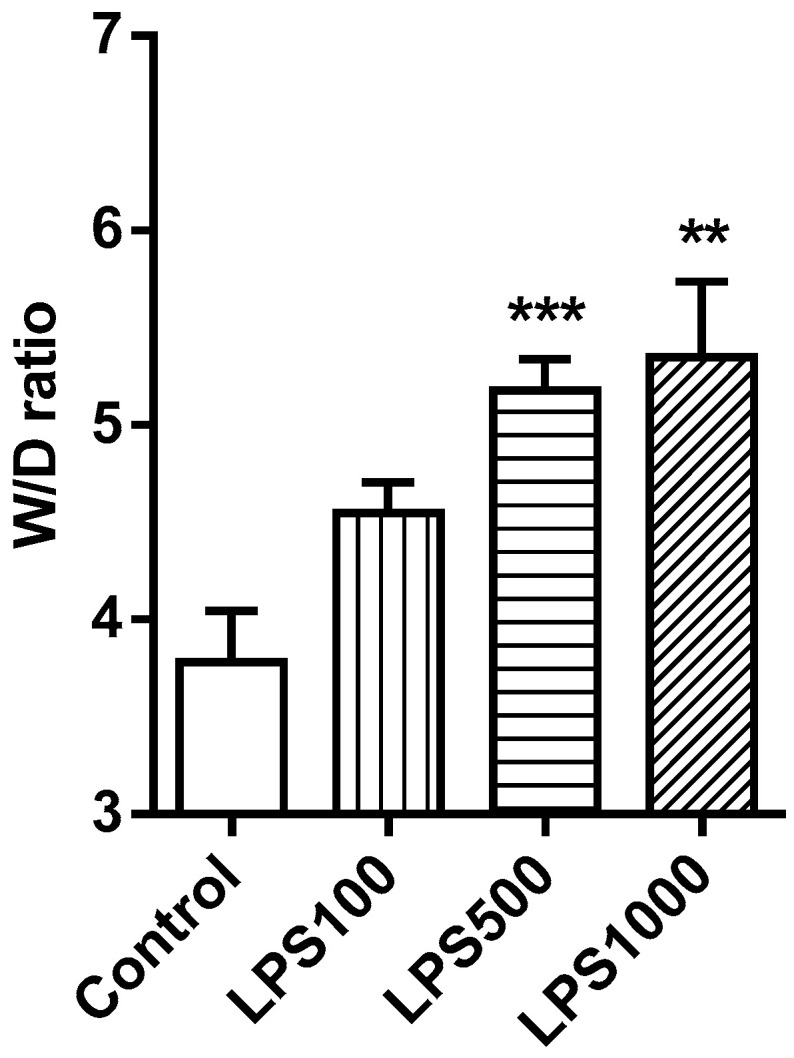
Lung edema formation at the end of the experiment. Values represent the W/D (wet/dry) weight ratio of lung tissue. Control vs. LPS-treated groups ** *p* < 0.01, *** *p* < 0.001.

Administration of LPS 500 and 1000 µg/kg b.w. significantly increased W/D weight ratio in comparison to control (LPS500 and LPS1000 vs. Control *p* < 0.001 and *p* < 0.01, respectively). The effect of LPS was dose-dependent (LPS500 vs. LPS100 *p* < 0.01 and LPS1000 vs. LPS100 *p* < 0.05) (Figure 1).

#### 2.1.2. Inflammatory Markers and Vascular-Specific Biomarker

**Figure 2:**
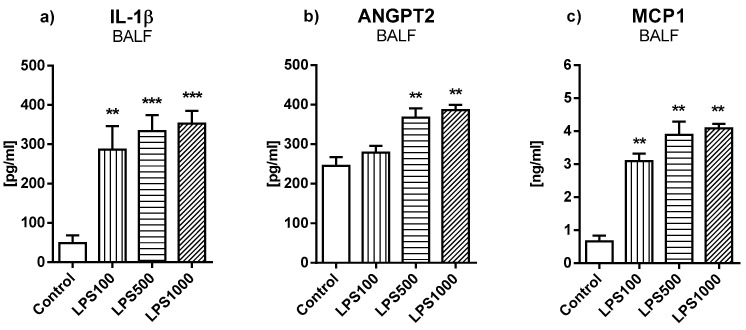
Levels of (**a**) Interleukin 1β (IL-1β), (**b**) angiopoietin 2 (ANGPT2), and (**c**) monocyte chemotactic protein 1 (MCP1) in bronchoalveolar lavage fluid (BALF). Control vs. LPS-treated groups ** *p* < 0.01, *** *p* < 0.001.

Inflammatory and vascular-specific markers were evaluated in bronchoalveolar lavage fluid (BALF) at the end of the experiments (Figure 2). Administration of LPS 100 µg/kg evoked an increase in IL-1β and MCP1 (both *p* < 0.01 vs. control group). However, the instillation of LPS at higher doses 500 µg/kg and 1000 µg/kg led to an increase in all investigated markers compared to the control group (Control vs. LPS500 and LPS1000 IL-1β *p* < 0.001, ANGPT2 and MCP1 *p* < 0.01).

#### 2.1.3. Oxidative Damage of the Lungs

**Figure 3:**
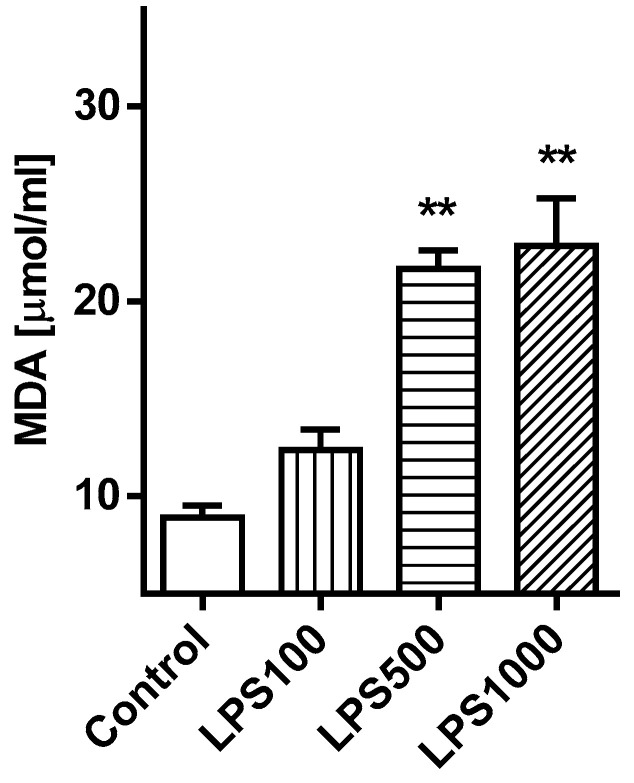
The values of malondialdehyde (MDA) in lung tissue. Control vs. LPS-treated groups ** *p* < 0.01.

Oxidative damage of lipids detected by the formation of malondialdehyde (MDA) in tissue homogenate was increased in groups LPS500 and LPS1000 compared to Control (both *p* < 0.01). In comparison with all LPS-treated groups differences in the MDA level between LPS500 vs. LPS100 and LPS1000 vs. LPS100 (both *p* < 0.05) were found (Figure 3).

#### 2.1.4. Total Leukocyte Count

**Figure 4:**
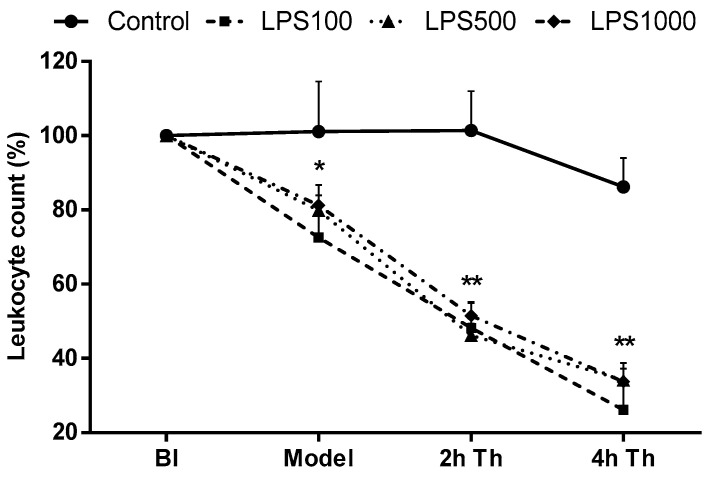
Total leukocyte count in arterial blood before instillation (BI) of saline (Control) or different doses of lipopolysaccharide (LPS100, 500, and 1000 µg/kg), 1 h after instillation (Model) and therapy (Th) administration during 4 h of experiment, expressed as a percentage (%). Control vs. LPS-treated groups * *p* < 0.05, ** *p* < 0.01.

Prior to LPS or saline administration, no significant differences in total leukocyte count in the arterial blood between all groups were determined. Administration of LPS led to a significant decrease in the total leukocyte count (Model; LPS groups vs. Control, all *p* < 0.05). At 2 and 4 h after the therapy, the total leukocyte count decreased in all animals with LPS compared to the control group (all LPS groups *p* < 0.01 vs. Control). No significant differences among the three LPS-instilled (100, 500, 1000) groups throughout the whole experiment were present (Figure 4).

#### 2.1.5. Gene Expression of Surfactant Proteins SP-A, SP-B, SP-C, and SP-D

According to previous results, gene expression of surfactant proteins was evaluated only in LPS500 and LPS1000 groups and control.

**Figure 5:**
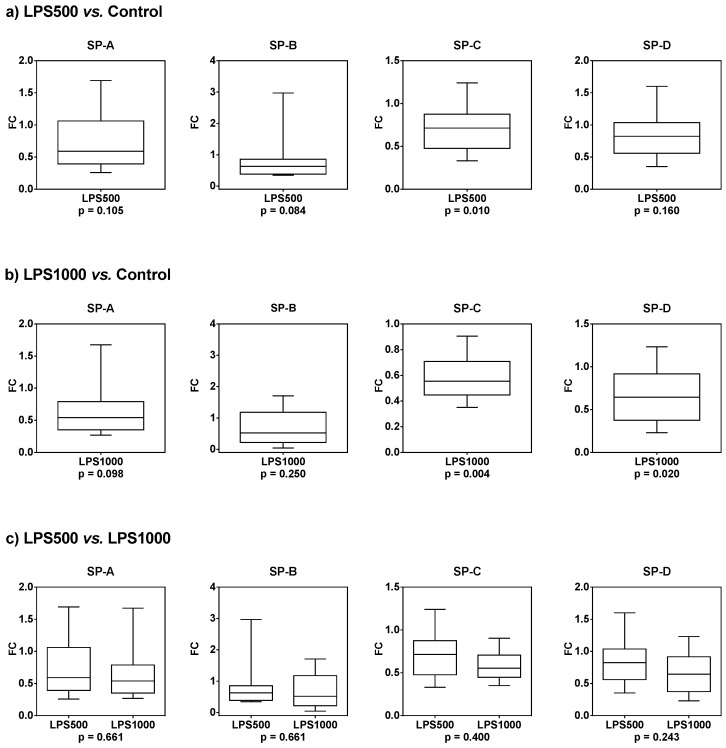
The gene expression of SPs after treatment with lipopolysaccharide (LPS) at a concentration of (**a**) 500 µg/kg or (**b**) 1000 µg/kg compared to the control group. (**c**) Comparison of SP gene expression after treatment with 1000 and 500 µg/kg LPS.

Compared to the control group, the gene expression of all SPs was downregulated in groups treated with 500 µg/kg LPS (Table 1, Figure 5a), and LPS at dose 1000 µg/kg even further potentiated this effect (Table 1, Figure 5b). There was no significant difference between 500 and 1000 µg/kg LPS (Table 1, Figure 5c).

### 2.2. Second Series

#### 2.2.1. Lung Oedema Formation

**Figure 6:**
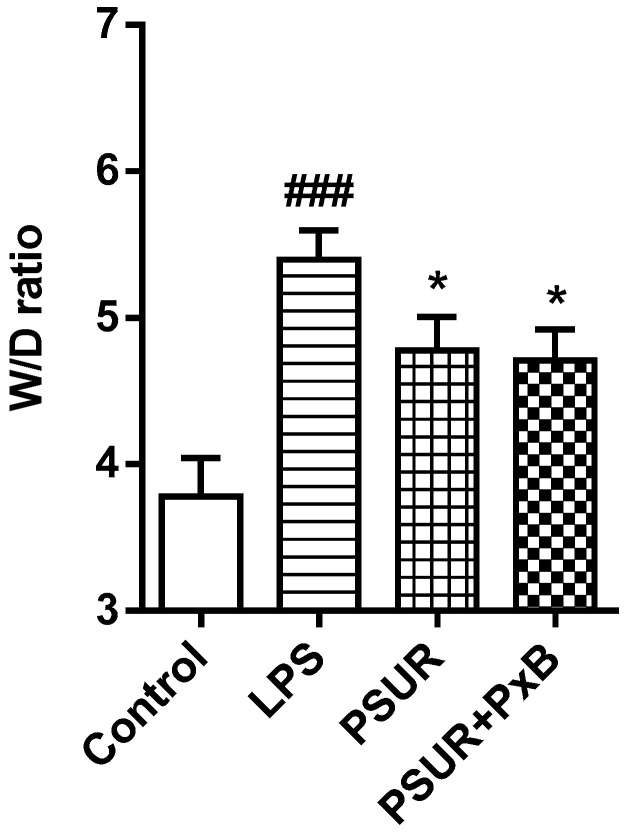
Lung oedema formation at the end of the experiment. Values represent W/D (wet/dry) weight ratio of lung tissue. Control vs. LPS ### *p* < 0.001, LPS vs. PSUR, LPS vs. PSUR + PxB, * *p* < 0.05.

In comparison to the control, W/D weight ratio significantly increased in the LPS-instilled group (Control vs. LPS *p* < 0.001). Administration of PSUR and PSUR + PxB reduced lung W/D weight ratio vs. LPS group (both *p* < 0.05) with no significant differences between groups receiving surfactant with or without PxB (Figure 6).

#### 2.2.2. Cytokines and Inflammatory Markers

**Figure 7:**
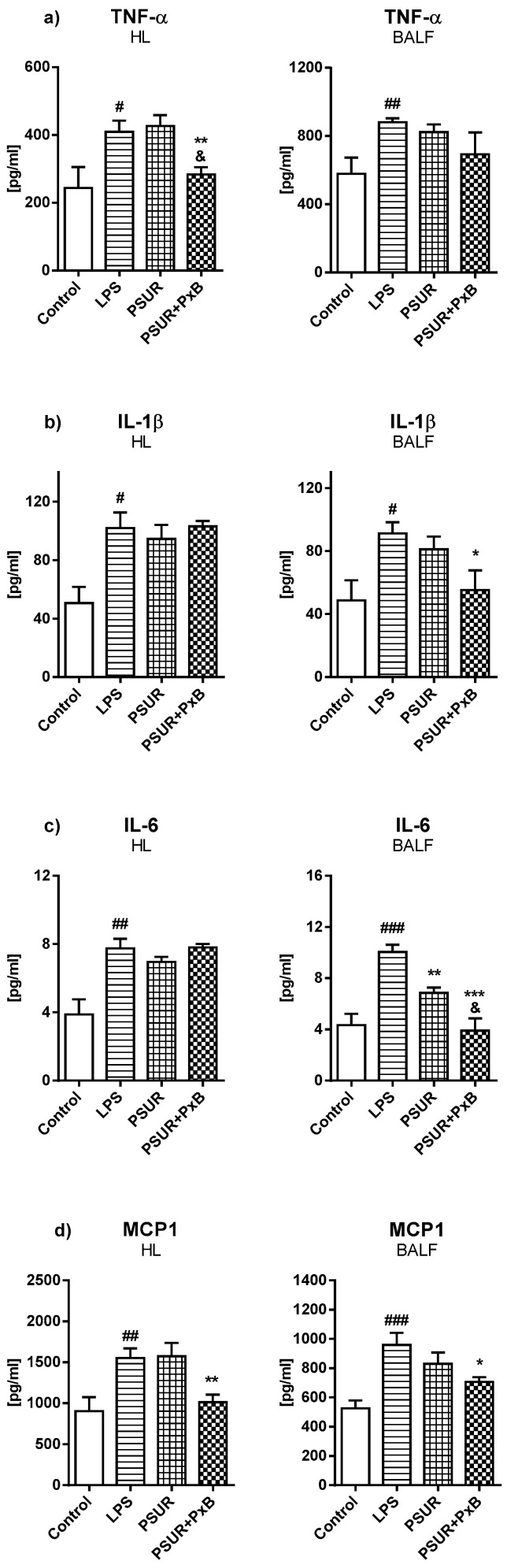
Levels of (**a**) tumor necrosis factor alpha (TNFα), (**b**) interleukin 1β (IL-1β), (**c**) interleukin 6 (IL-6), and (**d**) monocyte chemotactic protein 1 (MCP1) in homogenized lung (HL) and in bronchoalveolar lavage fluid (BALF). Control vs. LPS # *p* < 0.05, ## *p* < 0.01, ### *p* < 0.001, PSUR vs. LPS, PSUR + PxB vs. LPS * *p* < 0.05, ** *p* < 0.01, *** *p* < 0.001, PSUR vs. PSUR + PxB ^&^ *p* < 0.01.

Levels of cytokines and inflammatory markers were evaluated at the end of the experiment both in the homogenized lung (HL) and in bronchoalveolar lavage fluid (BALF). The levels of all investigated markers were significantly higher in the group with LPS and no further treatment (vs. Control, all *p* < 0.05 to 0.001). Administration of PSUR caused a decrease only in IL-6 in BALF compared to the LPS group (*p* < 0.01). On the other hand, PSUR + PxB induced reduction of TNFα and MCP1 in HL (both *p* < 0.01), and IL-1β, MCP1 (both *p* < 0.05), and IL-6 in BALF (*p* < 0.001) compared to the LPS group. Moreover, PSUR + PxB significantly decreased TNF-α in HL and IL-6 in BALF compared to PSUR (both *p* < 0.01) (Figure 7). Evaluation of CINC1 showed an elevation in the LPS group compared to the control in HL (*p* < 0.01) while the administration of PSUR and PSUR + PxB had no effect (data not shown, both *p* > 0.05). The level of caspase 3 (CASP3) did not change significantly in HL or in BALF (data not shown, *p* > 0.05).

#### 2.2.3. Oxidative Damage of the Lungs

**Figure 8:**
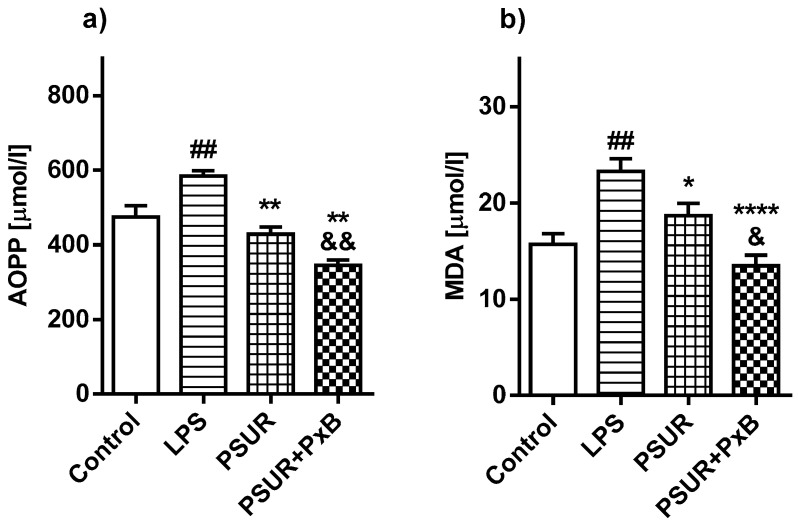
The values of (**a**) AOPP (Advanced Oxidation Protein Products) and (**b**) malondialdehyde (MDA) in lung tissue. Control vs. LPS ## *p* < 0.01; PSUR vs. LPS; PSUR + PxB vs. LPS * *p* < 0.05, ** *p* < 0.01, **** *p* < 0.0001; PSUR vs. PSUR + PxB ^&^ *p* < 0.05, ^&&^ *p* < 0.01.

Oxidative damage of proteins in homogenized lung tissue (Advanced Oxidation Protein Products; AOPP) was higher in the LPS-instilled group compared to the control group (LPS vs. Control *p* < 0.01). Administration of PSUR or PSUR + PxB significantly reduced the oxidative damage of proteins compared to the LPS group (PSUR vs. LPS *p* < 0.01; PSUR + PxB vs. LPS *p* < 0.01) with more pronounced reduction in animals receiving surfactant enriched with PxB (PSUR vs. PSUR + PxB *p* < 0.01; Figure 8a).

Oxidative damage of lipids was higher in the LPS-instilled group compared to controls (LPS vs. Control *p* < 0.01). The level of MDA in both treated groups was significantly reduced after surfactant alone or with PxB in comparison with the LPS group (PSUR vs. LPS *p* < 0.05; PSUR + PxB vs. LPS *p* < 0.0001). The effect of the surfactant/PxB mixture on oxidative lipid damage was stronger than the effect of surfactant alone (PSUR vs. PSUR + PxB *p* < 0.05; Figure 8b).

#### 2.2.4. Total Leukocyte Count

**Figure 9:**
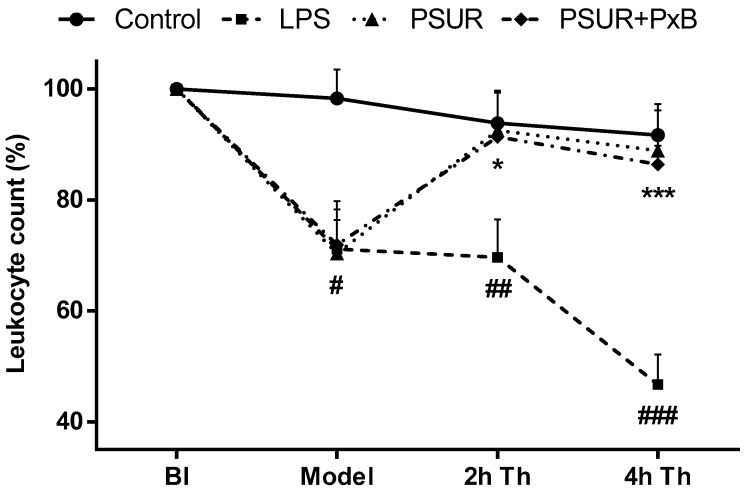
Total leukocyte count in arterial blood before instillation (BI) of saline (Control) or lipopolysaccharide (LPS), 1 h after instillation (Model) and therapy (Th) administration during 4 h of experiment, expressed as a percentage (%). Control vs. LPS #*p* <0.05, ## *p* <0.01, ### *p* <0.001, PSUR vs. LPS, PSUR + PxB vs. LPS * *p* < 0.05, *** *p* < 0.001.

At the beginning of the experiment, there were no differences in leukocyte count in the arterial blood between the groups. Instillation of LPS induced a significant decrease in the total count of leukocytes in all groups (all *p* < 0.05 vs. Control). In the second hour after the treatment, a decrease in leukocyte count was observed in the LPS group (*p* < 0.01 vs. Control) with the biggest drop at the end of the experiment (*p* < 0.001 vs. Control). The therapy led to an increase in the total leukocyte count in both surfactant-treated groups in 2 h of therapy almost to the control level (both *p* < 0.05 vs. LPS), keeping the trend until the end of the experiment (4 h after therapy both *p* < 0.05 vs. LPS) (Figure 9).

#### 2.2.5. Gene Expression of Surfactant Proteins SP-A, SP-B, SP-C, and SP-D

**Figure 10:**
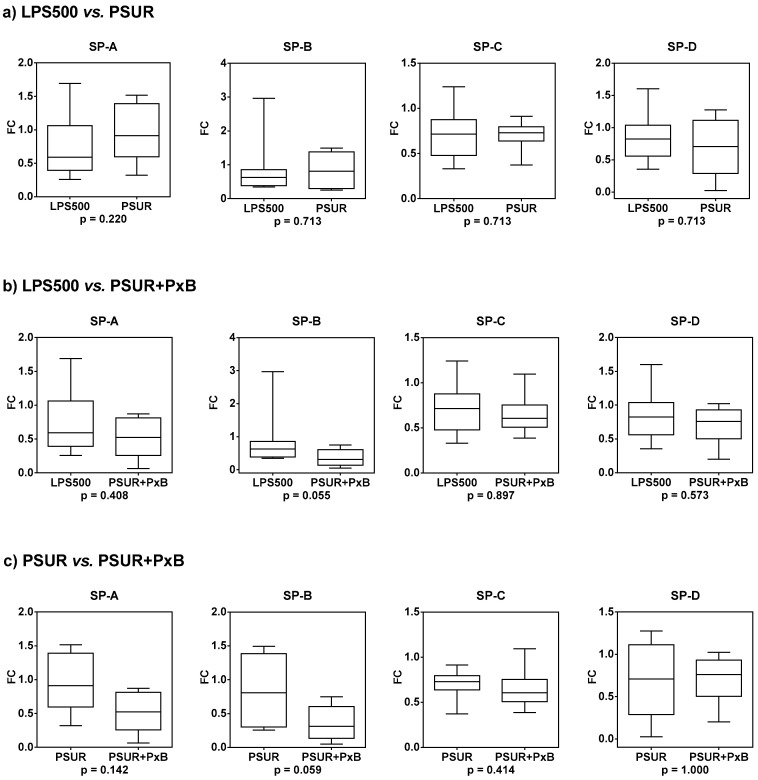
The gene expression of SPs after treatment with (**a**) poractant alfa (PSUR) or (**b**) poractant alfa with polymyxin B (PSUR + PxB) compared to the group treated with LPS 500 µg/kg. (**c**) Comparison of SP gene expression after treatment with poractant alfa alone or in combination with PxB.

Compared to the group treated with 500 µg/kg LPS, the gene expression of SP-A and SP-B increased after treatment with PSUR, the gene expression of SP-C did not change and the gene expression of SP-D slightly decreased (Table 2, Figure 10a). The gene expression of all SPs was further downregulated after treatment with PSUR combined with PxB (Table 2, Figure 10b). Compared to the group treated with PSUR alone, there was a trend to decrease gene expression of SP-A and SP-B with PSUR/PxB. The gene expression of SP-C and SP-D almost did not change after combined therapy (Table 2, Figure 10c).

## 3. Discussion

The aim of the study was to prove the hypothesis that intratracheal administration of surfactant/polymyxin B mixture reduces LPS-induced lung injury more effectively than surfactant treatment alone in mechanically ventilated adult rats. Since surfactant specific proteins (SPs) are expressed at relatively high levels in alveolar type II (ATII) cells and are crucial for alveolar homeostasis, an important objective was to evaluate the impact of this treatment on the expression of SPs encoding genes.

The harmful effect of LPS on pulmonary capillary permeability and surfactant has been known for many decades [19]. It was stated that the complexing of endotoxin with a pulmonary surfactant may contribute to pathological changes observed in pneumonia and these changes have been attributed to surfactant alterations [20]. LPS interacts with all SPs [21,22,23] and phospholipids which promote destabilization of surfactant film [10]. LPS-induced lung injury is a widely used animal model to test treatment possibilities, however, literary data on the appropriate dose of LPS vary. In different studies, LPS was intratracheally (*i.t*.) administered at doses between 5 to 5000 μg/kg b.w. [24,25,26]. In the first series of experiments, we tested three different doses of LPS (100, 500, and 1000 μg/kg b.w.) in order to find out the optimal one to induce significant lung injury at a 100% survival rate and thus is suitable for further experiments. This goal has been reached at an LPS dose of 500 μg/kg which led to lung damage and edema formation in accordance with other studies [25,27]. There was an increase in the level of lipid peroxidation products in the lung tissue, an increase in the level of biomarkers (IL-1β, ANGPT2, and MCP-1) in BALF, as well as a decrease in total white blood cell count during the experiment indicative of inflammatory cell redistribution between the lung and systemic circulation.

The mechanism responsible for endotoxin-induced lung damage is believed to be LPS binding to TLR4 receptor complex present on cell membranes, increased activation of NF-κB and subsequent proinflammatory and pro-oxidative pathways [28] followed by ARDS-like tissue damage [25,29]. Acute inflammation induced by LPS contributes to increased permeability of the alveolar-capillary membrane [30], which in our experiments was evident from the change in the ratio of wet/dry lung tissue weight indicating pulmonary edema.

In addition to the above-mentioned changes, LPS at a dose of 500 µg/kg had a trend towards the decreased expression of all genes encoding SPs which was significant for SP-C, while for LPS at a dose of 1000 µg/kg a decrease in gene expression was significant for SP-C and SP-D. These results correspond to studies in animal models of acute lung injury, which have reported reduced SPs mRNA levels [31,32,33,34] and findings in BALF of ARDS patients [35]. The reduction of SPs caused by bacterial products is thought to be mediated through IL-1 and TNF-α [36,37]. Moreover, LPS has been shown to reduce the viability of ATII cells [38,39] and modulate gene expression of all SPs [40]. Thus, the mechanism of reduced SPs gene expression is complex and may be related to increased levels of proinflammatory mediators, direct or indirect ATII cells damage, and reduced number of ATII cells due to their apoptosis and necrosis as the response to LPS [40]. Altogether, this leads to changes in ATII cell metabolism and consequently in the secretion of SPs and other surfactant components.

The in vitro studies clearly show that LPS binds to the pulmonary surfactant and prevents it from reaching low surface tension even at an LPS concentration of 1% [10]. In vivo, endotoxin-induced lung damage can be favorably affected by *i.t.* administration of exogenous surfactant. In our experiments, administration of poractant alfa alone reduced edema formation, lipid and protein oxidation, IL-6 production in BALF, and prevented white blood cell redistribution. Exogenous surfactant was administered at a dose of 50 mg of phospholipids/kg of body weight which is lower than the clinically recommended dose (200 mg/kg). A lower dose of surfactant was used in order to demonstrate the positive effect of the surfactant/PxB mixture. At a lower surfactant dose, the ability of PxB to “improve” surfactant and protect it against LPS inactivation could be better manifested.

In the past, the favorable effect of surfactant treatment was confirmed for both modified porcine and synthetic surfactants. Kinniry et al. [41] used synthetic preparation based on the KL4-peptide in a mouse model of LPS-induced lung injury. Mittal and Sanyal [32] reported the positive effect of the natural surfactant which was more pronounced than the effect of the synthetic preparation enriched with tyloxapol, which is no longer used clinically. In both studies, the effect of surfactant treatment was verified late, after 72 or 24 h, respectively. In the only existing study with modified porcine surfactant, poractant alfa was administered to spontaneously breathing rats with LPS-induced lung damage resulting in reduced mortality [42]. In our model based on mechanically ventilated animals, the clinical situation of ARDS patients may be better reflected and enable the evaluation of the acute effects of surfactant treatment within a few hours of administration.

In addition, administration of poractant alfa had a favorable effect on the regulation of the SPs gene expression as there was a trend to increase SP-A and SP-B gene expression in animals exposed to 500 µg/kg LPS. Literary data on the expression of SPs other than SP-A [32] in relation to exogenous surfactant therapy are lacking. The trend for the increased expression of SPs after poractant alfa administration may be the result of suppressed inflammation, as demonstrated by reduced levels of cytokines and proinflammatory mediators, as well as damage or death of ATII cells due to less oxidative stress. It makes ATII cells able to continue in the production of pulmonary surfactant and thus help to combat the negative effects of LPS. Especially, pulmonary collectins SP-A and SP-D are an important part of host defense against respiratory pathogens and allergens [43] and their expression can increase in order to reduce the adverse effect of LPS on the lungs.

Pulmonary surfactant can be inactivated by inhibitors that enter the lung. Surfactant inactivation can be overcome by the addition of antimicrobial peptide PxB to existing surfactant preparations [44]. PxB binds *E. coli* LPS and prevents the activation of inflammatory pathways [45]. Moreover, it is able to mimic the properties of SP-B [8] and increase the resistance of surfactant preparations. Its beneficial effect was observed in vitro [10] but also in vivo when the surfactant/PxB mixture has been effective in animals with ARDS due to serum albumin leakage into the alveolar space [9] or in newborn rabbits with LPS-induced lung injury [46]. Therefore, the surfactant/PxB mixture appears to be a suitable treatment for Gram-negative lung infections. In our rat model of LPS-induced lung injury, this mixture had an even more pronounced effect on pulmonary edema, oxidative stress, and inflammatory parameters than treatment with poractant alfa alone. Despite the positive effects of poractant alfa/PxB treatment, there was no improvement in the level of SPs gene expression. On the contrary, in comparison with LPS-instilled animals, the gene expression of SP-A, SP-B, and SP-D was even more reduced.

Polymyxins are generally considered to be toxic [11]. As for the lungs, information on possible toxicity is limited to two studies in which PxB induced oxidative stress in alveolar epithelial cells and induced their apoptosis [15,17]. Nephrotoxicity induced by polymyxins may be the result of their extensive reabsorption by renal tubular cells mediated by the oligopeptide transporter PEPT2 [47]. As PEPT2 is highly expressed in the airway epithelium [48], it might have a significant effect on PxB toxicity in the lungs. When PxB enters the cell, it can destabilize intracellular membranes and induce changes in their permeability, leading to the overproduction of ROS. The excessive amounts of ROS reduce the activity of antioxidant enzymes and triggers signaling cascades leading to cell damage and subsequent cell death [49]. Both external (receptor) and internal (mitochondrial) apoptotic pathways are involved in PxB-induced apoptosis of alveolar epithelial cells [15]. This is evidenced by increased expression of the Fas ligand (FasL) and activation of caspases 3, 8, and 9 in A549 cells after PxB treatment. The hypothesis that ROS could be an important factor by which PxB induces apoptosis of ATII cells is supported by studies in which administration of antioxidants or ROS scavengers such as ascorbic acid and NAC reduced oxidative stress levels and protected renal tubular cells from apoptosis induced by polymyxin E [50,51]. The possible toxic effect of PxB on ATII cells could explain the trend of the reduced SPs gene expression after administration of the surfactant/PxB mixture.

Negative effects of PxB on ATII cells were suppressed in the mixture with surfactant [17] due to the direct interaction of PxB with surface films, allowing bonding between two phospholipid monolayers. The release of PxB from the surfactant lipoprotein complex is delayed, reducing its peak concentrations. On the contrary, PxB appeared to have an adverse effect on ATII cells, manifesting by the tendency to reduce SPs gene expression after treatment with surfactant/PxB mixture, whereas SPs gene expression increased after treatment with surfactant alone. However, the molecular mechanisms responsible for these changes are yet unknown and should be the subject of further study.

## 4. Materials and Methods

### 4.1. Animals

For all experiments, 50 adult male rats (Wistar) with bodyweight (b.w.) 330 ± 20 g have been used. Animals were supplied by VELAZ Animal Breeding Station in the Czech Republic and were housed five per cage in transparent plastic cages with bedding, enhanced by plastic tubes, at a temperature of 20–24 °C and 55 ± 10% and under 12/12 h light/dark cycle. Rats were fed a standard diet (VELAZ) once per day according to the weight range and the access to water was ad libitum.

### 4.2. Chemicals

#### 4.2.1. Lipopolysaccharide (LPS)

Characteristic component of the cell wall of Gram-negative bacteria, purified lyophilized phenol extract of *Escherichia coli* (O55:B5, Santa Cruz Biotechnology, Inc., Dallas, TX, USA) was dissolved and diluted in sterile saline.

#### 4.2.2. Modified Porcine Surfactant

Modified porcine surfactant poractant alfa (Curosurf^®^; Chiesi Pharmaceutici, Parma, Italy) provided in the concentration of 80 phospholipids (PLs) mg/mL was administered undiluted intratracheally (i.t.) at a dose of 50 mg PL/kg (0.625 mL/kg b.w.).

#### 4.2.3. Polymyxin B (PxB)

The stock solution of PxB (polymyxin B sulfate; AppliChem GmbH, Darmstadt, Germany) was prepared in Aqua Pro injection at concentrations of 5 mg/mL. It was added to exogenous surfactant preparation at a concentration of 1% (by mass of surfactant PLs, *w*/*w*) and the mixture was gently shaken and incubated for 30 min at 37 °C before i.t. instillation.

### 4.3. First Series

Since the literary data on LPS dose for lung injury induction by intratracheal administration diverged, the aim of the first part of the study was to determine the appropriate dose to induce lung injury so that the animals survived the desired 4 h period of observation but had quantifiable lung injury. In this first series, 24 adult male Wistar rats were used. To induce lung injury, animals were instilled by LPS at a dose of 100, 500, and 1000 µg/kg b.w. in a volume of 2.2 mL/kg b.w. The control group received saline in the same volume. LPS or saline were heated to 37 °C and administered through a tracheal cannula with a thin short catheter on the syringe cone while positioning the animal to the right and to the left. The animals were divided as follows (in each group, *n* = 6): control group with saline (control), LPS group at 100 µg/kg (LPS100), LPS group at 500 µg/kg (LPS500), and LPS group at 1000 µg/kg (LPS1000) and mechanically ventilated as described below.

### 4.4. Second Series

Based on the results of the previous series of experiments, for testing the therapeutic effect of surfactant with/without PxB the LPS dose of 500 µg/kg b.w. was used. In the second part of the study, 26 adult male Wistar rats were divided into four groups. Three groups were given LPS at 500 µg/kg and after the development of lung injury were further ventilated with no treatment (LPS group; *n* = 6); or received poractant alfa (50 mg PLs/kg b.w.) (PSUR group; *n* = 7) or poractant alfa at the same dose mixed with Polymyxin B (1% by mass of surfactant PLs, *w/w*) (PSUR + PxB group; *n* = 7). Surfactant, or its mixture with PxB, was given through a thin cannula, and during instillation, ventilatory parameters were adjusted to keep tidal volume (Vt) at 6 mL/kg [52]. Control animals received saline in the same volume instead of LPS (Control group; *n* = 6).

### 4.5. The Study Design

The protocol of our experiments (Project identification code VEGA 1/0055/19) was approved by the local ethics committee of Jessenius Faculty of Medicine, Comenius University and National Veterinary Board of the Slovak Republic. The protocol follows EU Directive 2010/63/EU for animal experiments and comply with the ARRIVE guidelines.

The animals were anesthetized with i.p. administration of ketamine (90 mg/kg) and xylazine (10 mg/kg), followed by i.v. infusion of ketamine (60 mg/kg/h). The animals were tracheotomized and an endotracheal tube was inserted. Catheters were placed into the artery femoralis for blood sampling and monitoring blood pressure and vein femoralis for continuous anesthesia infusion. After cessation of spontaneous breathing by pipecuronium bromide (0.3 mg/kg/30 min i.v.; Arduan, Gedeon Richter, Hungary), animals were ventilated by a volume-controlled ventilator (Harvard Apparatus, Inspira ASV, Massachusetts, USA) with a tidal volume (V_T_) of 6 mL/kg, respiratory rate (RR) of 50 breaths per minute (bpm), fraction of inspired oxygen (FiO_2_) of 0.4, inspiration time (Ti) of 50 %, and positive end-expiratory pressure (PEEP) of 0.3 kPa. After 15 min of stabilization, LPS or saline at a dose of 2.2 mL/kg were instilled i.t. while the animals were positioned to the right and left. Lung injury was determined as a reduction in dynamic compliance >30% or a decrease in PaO2/FiO2 (a ratio between arterial oxygen partial pressure and fraction of inspired oxygen) <26.7 kPa which denotes moderate lung injury and it took approximately one hour from instillation for the model to be established. Immediately after the determination of lung injury, all animals were administered by the treatment and further ventilated for an additional 4 h. During observation, measurements of gas exchange (PaO_2_ and PaCO_2_) and pH were made in arterial blood by gas analyzer (Rapidlab TM348 Bayer Diagnostics, Germany). Body temperature was continuously measured and maintained while placing the animals on the heating plate of the animal temperature controller (Physitemp Instruments, Inc., TCAT-2LV, Clifton, NJ, USA) inserted 2–3 cm into the anus.

At the end of the experiment, animals were overdosed by anesthetics and the thorax was opened. The trachea was cut just below the larynx. The lungs still connected to the heart were excised. The left lung lobes were lavaged with sterile saline (2 × 10 mL/kg b.w.). The recovered bronchoalveolar lavage fluid (BALF) was centrifuged (1500 rpm for 15 min). The supernatant was removed and immediately frozen at −70 °C prior to analysis. A piece of the right lung was used for lung edema formation, and other pieces of the right lung were further processed. One part was stored immediately in RNAlater (RNAlater^TM^, R0901, Thermo Fisher Scientific, Waltham, MA, USA) for further PCR analyses. After 24 h of RNAlater penetration to the tissues at room temperature, samples were moved to −70 °C. Another part of lung tissue was washed in cold phosphate-buffered saline (PBS) (0.01 M) and weighed. It was cut into small pieces and homogenized in PBS by homogenizer (Polytron^®^ PT 1200, Kinematica AG, Luzern, Switzerland) to a final concentration of 10% (weight/volume). The suspension was subjected to two freeze-thaw cycles to further break the cell membranes. After that the homogenate was centrifuged (15,000 rpm for 15 min) and the supernatant was removed and stored at −70 °C for biochemical analyses.

### 4.6. Assays

The concentrations of malondialdehyde (MDA; OxiSelect™ TBARS Assay Kit (MDA, Cell Biolabs Inc., San Diego, CA, USA) and chloramine-T (OxiSelect™ AOPP Assay Kit, Chloramine-T, Cell Biolabs Inc., San Diego, CA, USA) were determined in the homogenized lung (HL) and expressed in µmol/L. Tumor necrosis factor-alpha (TNFα), interleukin 6 (IL-6), interleukin 1β (IL-1β), monocyte chemotactic protein 1 (MCP1), angiopoietin 2 (ANGPT2), caspase 3 (CASP3; all Cloud-Clone Corp., Houston, TX, USA), rat pulmonary surfactant-associated protein A (SP-A; Cusabio Biotech Co., Wuhan, China), and cytokine-induced neutrophil chemoattractant-1 (CINC-1, R&D Systems, Inc., Minneapolis, MN, USA) were evaluated using commercially available ELISA kits.

### 4.7. Evaluation of Lung Edema

Parts of the wet right lung were weighed before and after being dried at 60 °C for 24 h. Lung edema formation was expressed as wet/dry (W/D) lung weight ratio.

### 4.8. Total Leukocyte Count

Total leukocyte count was determined in arterial blood by veterinary hematologic analyzer Sysmex XT-2000i (Sysmex, Kungsbacka, Sweden) and expressed as a percentage of basal values.

### 4.9. Evaluation of Gene Expression of Surfactant Proteins by Quantitative Real-Time PCR

Total RNA was extracted from the frozen lung tissue stored at −70 °C in RNA prior to analysis using TRI Reagent^®^ (Molecular Research Center, Inc., Cincinnati, OH, USA) and transcribed into cDNA using a QuantiTect Reverse Transcription Kit (Qiagen, Düsseldorf, Germany) according to the manufacturer’s instructions. The expression of genes encoding surfactant proteins (*SFTPA, SFTPB, SFTPC,* and *SFTPD*) and ribosomal protein 13a (RPL13a) as a housekeeping gene was analyzed by quantitative real-time PCR on iCycler iQ™ Real-Time PCR Detection System (Bio-Rad, Hercules, CA, USA) using an SYBR Green detection protocol with uracil-N-glycosylase (UNG) (Qiagen, Düsseldorf, Germany) implementing 100 ng of each cDNA. The amplification program consisted of 2 min of UNG pretreatment at 50 °C followed by a 15 min initial activation step at 95 °C and 40 cycles of 15 s denaturation at 94 °C, 30 s annealing at 55 °C, and 30 s extension at 72 °C. Primer sequences used according to Schmiedl et al. [53] are shown in Table 3. Data were analyzed by the standard 2^−ΔΔCt^ method.

### 4.10. Statistical Analysis

The statistical analysis except surfactant proteins gene expression was performed with GraphPad Prism 6.04. Between-group differences were analyzed using the Mann–Whitney test. A value of *p* < 0.05 was considered to be statistically significant. All data are shown as mean ± standard error of the mean (SEM).

The RT PCR Ct data were processed by an in-house R code, to obtain the fold change (FC) expression. Statistical analysis of the resulting data consisted of the visual data explorations by means of a boxplot. For FC comparing a treatment to control, the null hypothesis of the equality of the median population FC to 1 was tested by the Wilcoxon test. When comparing gene expression for two treatments, the FC was log-transformed and the null hypothesis of equality of the difference of log-FC to zero was tested. The hypothesis was tested by the Wilcoxon two-sample test. The Wilcoxon unpaired two-sample test is invariant to a monotone transformation, which permitted the extension of the statistical significance finding to FC itself. Findings with a *p*-value below 0.05 were considered statistically significant. All the analyses were performed in R (R Core Team, 2019) [54] ver. 3.6.1, with the aid of the library car (Fox and Weisberg, 2019) [55].

## 5. Conclusions

The mechanically ventilated adult rats with LPS-induced acute lung injury represent a clinically relevant model suitable for evaluation of acute treatment modalities. Administration of exogenous surfactant in rats with LPS mitigates inflammation and oxidative stress with a tendency to increase SPs gene expression reduced by LPS. Enrichment of exogenous surfactant with PxB potentiates the effect of surfactant therapy. However, because of the tendency to reduced SPs gene expression in the lungs after surfactant/PxB administration topical use of PxB should be considered with caution and should be subjected to further evaluation.

## Figures and Tables

**Table 1:** Median fold change (FC) and *p*-values for SPs gene expression after treatment with 500 and 1000 µg/kg LPS. Significant values are marked with an asterisk *.

	LPS500 vs. Control		LPS1000 vs. Control		LPS500 vs. LPS1000	
	Median FC	*p*-Value	Median FC	*p*-Value	Median FC	*p*-Value
SP-A	0.658	0.105	0.558	0.098	0.846	0.661
SP-B	0.627	0.084	0.623	0.250	0.916	0.661
SP-C	0.706	0.010 *	0.557	0.004 *	0.838	0.400
SP-D	0.824	0.160	0.645	0.020 *	0.702	0.243

**Table 2:** Median fold change (FC) and *p*-values for SP gene expression after treatment with PSUR alone or in combination with PxB.

	LPS500 vs. PSUR		LPS500 vs. PSUR + PxB		PSUR vs. PSUR + PxB	
	Median FC	*p*-Value	Median FC	*p*-Value	Median FC	*p*-Value
SP-A	1.374	0.220	0.798	0.408	0.572	0.142
SP-B	1.151	0.713	0.508	0.055	0.437	0.059
SP-C	1.022	0.713	0.950	0.897	0.894	0.414
SP-D	0.822	0.713	0.866	0.573	0.985	1.000

**Table 3:** SP-gen-specific sequences for the detection of SP-A, SP-B, SP-C, SP-D, and the housekeeping gene ribosomal protein L13a (RPL13a). (**F**) Forward and (**R**) reverse primer; (**bp**) base pair [53].

Gene	Primer Sequence Forward and Reverse Primer	Size PCR Product (bp)
SFTPA	F5´-CTAAGTGCTGCCCTCTGACC-3´ R5´-AGGAGCCATACATGCCAAAC-3´	247
SFTPB	F5´-CTGTGCCAAGAGTGTGAGGA-3´ R5´-CAAGCAGCTTCAAGGGTAGG-3’	124
SFTPC	F5´-CAGCTCCAGGAACCTACTGC-3´ R5´-CTCTCCACACAAGGTGCTCA-3´	218
SFTPD	F5´-ATGGCCAAAGTGTTGGAGAC-3´ R5´-CGTGCCCACATCTGTCATAC-3´	194
RPL13a	F5´-CCCTCCACCCTATGACAAGA-3´ R5´-TTCCGGTAATGGATCTTTGC-3´	186
